# Exosomes in the Pathogenesis, Progression, and Treatment of Osteoarthritis

**DOI:** 10.3390/bioengineering9030099

**Published:** 2022-02-27

**Authors:** Yishu Fan, Zhong Li, Yuchen He

**Affiliations:** 1 Department of Neurology, Xiangya Hospital, Central South University, Changsha 410008, China; fanyishu@csu.edu.cn; 2 Department of Orthopaedic Surgery, University of Pittsburgh School of Medicine, Pittsburgh, PA 15219, USA; 3 Department of Neurobiology, University of Pittsburgh School of Medicine, Pittsburgh, PA 15213, USA; 4 Department of Orthopaedics, Xiangya Hospital, Central South University, Changsha 410008, China

**Keywords:** osteoarthritis, exosome, extracellular vesicle, regenerative medicine, chondrocyte, cartilage injury

## Abstract

Osteoarthritis (OA) is a prevalent and debilitating age-related joint disease characterized by articular cartilage degeneration, synovial membrane inflammation, osteophyte formation, as well as subchondral bone sclerosis. OA drugs at present are mainly palliative and do not halt or reverse disease progression. Currently, no disease-modifying OA drugs (DMOADs) are available and total joint arthroplasty remains a last resort. Therefore, there is an urgent need for the development of efficacious treatments for OA management. Among all novel pharmaco-therapeutical options, exosome-based therapeutic strategies are highly promising. Exosome cargoes, which include proteins, lipids, cytokines, and various RNA subtypes, are potentially capable of regulating intercellular communications and gene expression in target cells and tissues involved in OA development. With extensive research in recent years, exosomes in OA studies are no longer limited to classic, mesenchymal stem cell (MSC)-derived vesicles. New origins, structures, and functions of exosomes are constantly being discovered and investigated. This review systematically summarizes the non-classic origins, biosynthesis, and extraction of exosomes, describes modification and delivery techniques, explores their role in OA pathogenesis and progression, and discusses their therapeutic potential and hurdles to overcome in OA treatment.

## 1. Introduction

Osteoarthritis (OA) is the most common form of arthritis, causing chronic joint pain, decline in joint function, physical disability, and impaired quality of life in the affected population [1]. According to data from the National Health Interview Survey (NHIS), doctor-diagnosed OA and other forms of arthritis affected 52.5 million American adults during 2011–2012, and by 2040, this number is expected to be increased by 49% [2], creating a considerable socioeconomic burden [3]. During OA progression, pathological changes have been reported to affect the whole joint, including cartilage degradation, osteophyte formation, abnormal subchondral bone remodeling, synovitis, meniscus and ligament degeneration, hypertrophy of the joint capsule, and increased vascularization, inflammatory infiltration, and fibrosis in the infrapatellar fat pad (IPFP) [4,5]. Risk factors of OA, including age, gender, genetic predisposition, obesity, inflammation, and excessive mechanical loading, increases the probability of OA occurrence and development [6]. With the combined effects of aging, obesity, and an increasing number of joint injuries in the global population, this burdensome syndrome is expected to become more prevalent [7]. 

Treatment strategies of OA are limited due to the lack of knowledge about OA pathogenesis. At present, no disease-modifying osteoarthritis drugs (DMOADs) are available to reverse or halt OA progression [8]. Pharmacological approaches, such as the use of non-steroidal anti-inflammatory drugs (NSAIDs), analgesics, and surgical interventions are current options to offer symptomatic relief [9]. However, these options are ineffective in repairing damaged articular cartilage, and are also challenged by relatively small effect sizes and uncertainty about their long-term efficacy and safety. These limitations hinder their clinical applications [10]. Considering that OA is a multifactorial disease with complex comorbid conditions, personalized treatment is essential to optimize outcomes [11]. To achieve this, researchers focus on developing personalized in situ intra-articular (IA) therapeutic options. IA drug delivery is superior to systemic administration with higher levels of efficacy and a lower risk of side effects. Different drug delivery systems have emerged to improve the local delivery of small molecules to joints [12]. Among them, exosomes, as a novel bio-cargo, have attracted significant attention in recent years.

**Figure 1:**
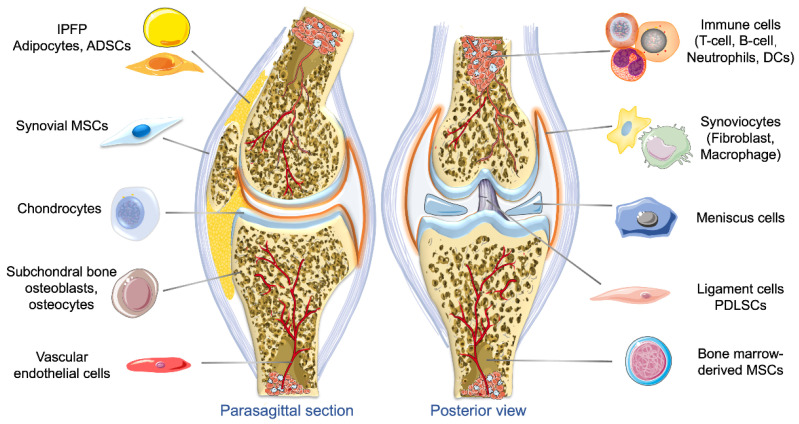
Tissue sources of exosomes in the knee joint. Exosomes are secreted by multiple types of cells of the joint, including adipocytes, adipose-derived stem cells (ADSCs), synovium-derived mesenchymal stem cells (MSCs), synovial fibroblasts and macrophages, chondrocytes, osteoblasts and osteocytes in the subchondral bone, vascular endothelial cells, immune cells such as T cells, B cells, and dendritic cells (DCs) meniscus cells, periodontal ligament cells, tenocytes, tendon stem cells, and bone marrow-derived MSCs. These exosomes are involved in the regulation of joint homeostasis, cell–cell communications, and the initiation and progression of OA.

Exosomes are a type of extracellular vehicles (EVs) with a diameter ranging between 30 and 150 nm, and a density of 1.13–1.19 g/mL [13]. These extracellular membrane-bound vesicles are able to work as cell-specific cargoes, which contain complex signaling molecules such as lipids, proteins, metabolites, nucleic acids, and cytosolic and cell-surface proteins [13]. Exosomes functions to mediate intercellular communications, and can be released into the extracellular environment by almost all types of cells through fusing plasma membrane and multivesicular bodies (MVBs) [14]. The biomedical applications of exosomes have been rapidly expanding in recent years because of their active roles in the function and pathophysiology of various body systems and potential in clinical therapeutics and diagnosis [15]. Diverse therapeutic payloads, such as DNAs, RNAs, antisense oligonucleotides, metabolites, chemotherapeutic agents, cytokines, and immune modulators, can be delivered to a target by engineered exosomes [16]. In OA related research, exosomes from multiple origins in the joint, such as tissue-specific mesenchymal stem cells (MSCs), chondrocytes, synovial fibroblasts (SFBs), osteoblasts, tenocytes, IPFP adipocytes, and platelet-rich plasma (PRP), have been detected and change with OA progression [17,18,19] (Figure 1). Herein we discuss the biosynthesis, origins, and contents of exosomes, and review their roles in OA pathogenesis, progression, and treatment.

## 2. Formation and Origin of Exosomes

The concept of ‘exosomes’ was first proposed in 1981 by Trams et al. [20]. In 1983, the currently defined exosomes were first identified in sheep reticulocytes and named by Johnstone et al. [21]. However, the widespread clinical applications were limited by the low yield for the production method used and unexpected therapeutic effects [22]. Besides, the function of exosomes is dependent on both the type and condition of the cells that they are released from, and thus varies a lot. To optimize the application, a comprehensive understanding of the generation, origins, and contents of exosomes is required.

### 2.1. Biogenesis of Exosomes 

**Figure 2:**
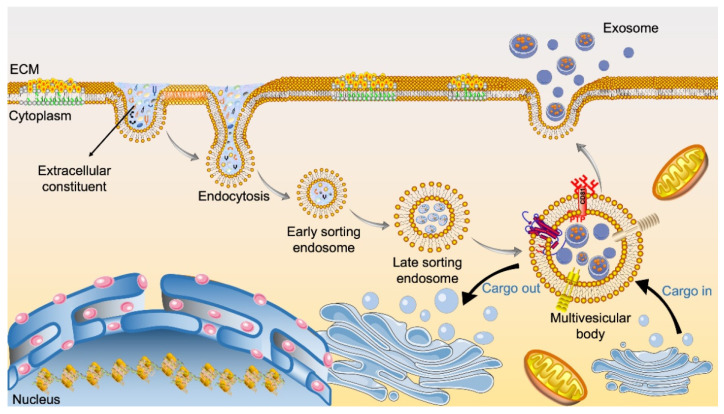
Scheme of the biogenesis of exosomes. Endocytosis and plasma membrane invagination facilitate the entry of cell surface proteins and extracellular components such as lipids, proteins, metabolites, ions, and small molecules into cells, leading to the formation of early sorting exosomes (ESEs). The ESEs then fuse with the endoplasmic reticulum (ER) and/or trans-Golgi network (TGN) and result in late sorting exosome (LSE) formation. A second invagination in the LSEs leads to the generation of multivesicular bodies (MVBs). MVBs can then either fuse with lysosomes for degradation or be transported to the plasma membrane and undergo exocytosis—a process resulting in exosome release. Exosomes, filled with various cellular components such as proteins, mRNAs, miRNAs, lipids, enzymes, and carbohydrates, are released through exocytosis after MVBs fuse with the cell membrane. Released exosomes can be further taken up by adjacent or remote cells in various ways, including receptor-mediated endocytosis and fusion with the plasma membrane of cells.

The detailed biological synthesis process of exosomes is shown in Figure 2. The cellular biogenesis process of exosomes begins with double invagination of the plasma membrane [23]. This is followed by the accumulation of bioactive substances in the early sorting endosomes (ESEs), such as lipids, proteins, small molecules, ions, and metabolites present in the extracellular space. The ESEs subsequently mature into late sorting endosomes (LSEs), a process regulated by endosomal sorting complex required for transport (ESCRT) proteins and others. After that, invagination of the limiting membrane of LSEs results in the formation of MVBs (also referred to as multivesicular endosomes) [16]. The MVBs can be degraded by fusing with autophagosomes or lysosomes; alternatively, MVBs fuse with the plasma membrane and release exosomes—vesicles containing the intra-endosome substances—to the extracellular space [23]. 

A key component of OA treatment is the efficient delivery of therapeutic molecules to targeted cells, especially to chondrocytes embedded in a dense extracellular matrix (ECM), which requires the use of biocompatible molecular transport vehicles. Exosomes exhibit unique features, such as high serum stability and strong penetration across biological barriers, which make them ideal cargoes for drug delivery in OA treatment [24]. However, pristine exosomes can experience fast clearance in the body and have weak cell-targeting abilities, resulting in unsatisfactory treatment outcomes. Therefore, bioengineered exosome-mediated delivery strategies, such as drug loading and surface modifications, have been explored to improve the cell-targeting property of exosomes [23]. For example, genetic engineering methods have been utilized to introduce specific proteins, such as ligands for receptors or antibodies against target cells, to the surface of exosomes to achieve precise delivery [25].

### 2.2. Origins of Exosomes and Their Roles in OA

Osteoarthritis is a whole-joint disease with pathological changes observed in all joint components [26]. Exosomes secreted by cells in joint tissues or from IA-injected therapeutic agents exhibit complex regulatory effects on the progression of OA [27]. MSCs, derived from tissues within the joint (e.g., subchondral bone, IPFP, and synovium) and elsewhere, represent the most widely studied sources of exosome production. In addition, exosomes have also been obtained from non-classic sources including, but not limited to, articular chondrocytes, adipocytes, osteoblasts, osteocytes, vascular endothelial cells, and PRP [28]. Exosomes derived from different origins exhibit varying effects. Some exosomes showed chondroprotective effects, while others, such as vascular endothelial cell (VEC) and OA chondrocyte-derived exomes, promoted OA progression. Detailed information and potential regulatory mechanisms of exosomes generated by different cells are listed in Table 1. In this section, the different exosome sources are discussed, with an emphasis on joint-related tissues and cells, followed by a description of their roles in OA.

#### 2.2.1. Exosomes Derived from Different Types of MSCs

MSCs possess multilineage differentiation potential and have been applied in IA injection therapies for OA treatment [57]. However, MSCs also face several limitations, including heterogeneity, inconsistent stemness, variable differentiation capacity, limited homing ability, and potential adverse effects, such as immune incompatibility, tumorigenicity, and chromosomal aberrations [58,59]. A growing body of evidence suggests that MSC-secreted exosomes should be credited for many of the previously reported regenerative properties of MSCs [60]. It was found that bone marrow MSC (BMSC)-derived exosomes can be endocytosed by chondrocytes. These exosomes showed capability in restoring the proliferation of chondrocytes, promoting ECM synthesis, and relieving knee OA pain [34]. Using MSC-derived exosomes to deliver mitochondrial-related proteins was reported to alleviate oxidative stress-induced damage and reverse mitochondrial dysfunction in degenerative OA cartilage [36]. MSC-derived exosomes containing a novel lncRNA KLF3-AS1 (KLF3 Antisense RNA 1; Ensembl: ENST00000440181) reversed the suppressive effects of miR-206 on the expression of G-protein-coupled receptor kinase interacting protein-1 (GIT1). It has been reported that GIT1 could promote the proliferation and inhibit the apoptosis of chondrocytes [61]. Thus, the lncRNA-KLF3-AS1/miR-206/GIT1 axis is possibly responsible for the chondroprotective effects of MSC-derived exosomes in OA [33]. 

In addition to BMSC, embryonic stem cell-derived MSCs (ESC-MSCs) [40], synovial MSCs (SMSC) [37,38], adipose-derived MSCs (ADSC) [21,62], umbilical cord mesenchymal stem cells (UC-MSCs) [42], periodontal ligament-derived stem cells (PDLSCs) [63], amniotic fluid stem cells (AFSCs) [46], and IPFP-MSCs [44,64,65] are other important origins of MSC-derived exosomes in OA treatment [38]. IA injection of ESC-MSC-derived exosomes facilitated the repair of osteochondral defects, maintained the chondrocyte phenotype, promoted cartilage formation, reduced matrix degradation, and impeded cartilage destruction both in vitro and in the destabilization of medial meniscus (DMM)-induced OA model in mice [40,66]. Mechanistically, these effects were achieved by promoting chondrocyte proliferation and migration, increasing collagen type II synthesis, and decreasing ADAMTS5 (A disintegrin and metalloproteinase with thrombospondin motifs 5) expression [40]. Exosomes obtained from miR-155-5p-overexpressing SMSC were used to treat OA chondrocytes and promoted their migration and proliferation, suppressed apoptosis, and enhanced the secretion of ECM; such exosomes also effectively prevented OA from occurring in mice undergoing cold water stimulation for 4 h/day over 20 days (which induced OA in the mice without exosome treatment) [37]. Recently, it was found that exosomes released by induced pluripotent stem cell (iPSC)-derived MSCs have a greater therapeutic effect compared with those from SMSCs [41]. IA injection of ADSC-derived exosomes showed an inhibitive effect on M1 macrophage infiltration into the synovium, significantly attenuating OA progression and preventing cartilage degeneration in both surgically induced (through DMM) mouse OA models and monosodium iodoacetate (MIA)-insulted rat joints [45]. Besides, ADSC-derived exosomes decreased the activity of senescence-related β-galactosidase in OA osteoblasts and the accumulation of γ H2AX foci, which were probably attributed to the protective effects on mitochondria [62]. Exosomes derived from UC-MSCs, which contained a high level of LncRNA H19, promoted chondrocyte proliferation and inhibited apoptosis in vitro; the exosomes also improved macroscopic assessment and relieved pain levels in a rat model of cartilage defect [42]. Furthermore, exosomes extracted from the conditioned medium of PDLSCs showed anti-inflammatory effects on chondrocytes, synoviocytes, and meniscus cells, mediating the inflammatory processes in various tissues in the joint [63]. AFSC-derived exosomes were found to increase pain tolerance and induce the restoration of hyaline cartilage with good surface regularity in an MIA-induced OA model [46]. An in vivo study showed that miR-100-5p-abundant IPFP-MSC-derived exosomes (IPFP-Exos) had chondroprotective effects and ameliorated gait abnormalities via inhibiting mTOR-autophagy pathway in an OA mouse model [44]. In vitro studies suggested that IPFP-Exos promoted chondrogenesis in periosteal cells via upregulating the expression of miR-221 and miR-145 and suppressing the production of proinflammatory cytokines [43]. Exosomes derived from kartogenin-pretreated IPFP-MSCs showed a stronger ability to induce stem cell chondrogenesis and promoted the proliferation of chondrocytes and the repair of articular cartilage defects both in vivo and in vitro [64]. Detailed information is summarized in Table 1.

#### 2.2.2. Exosomes Derived from Chondrocytes and Chondrogenic Progenitor Cells

Exosomes released by chondrocytes participate in the pathologic mineralization of OA cartilage and cell–cell communication [67,68], affecting cartilage maintenance and OA pathogenesis [19]. These exosomes have dual roles in OA, depending on the cell condition and cell types. Exosomes from healthy chondrocytes had high bioactivity in the elimination of mitochondrial dysfunction and restoration of immune reaction by regulating M2 macrophage penetration, thus delaying OA progression [31]. These chondrocyte-derived exosomes contained miR-8485, which inhibited the expression of glycogen synthase kinase (GSK)-3β and activated the Wnt/β-catenin pathway, promoting the chondrogenic differentiation of BMSCs [69]. On the contrary, exosomes derived from OA chondrocytes enhanced chondrocyte apoptosis, inhibited cell proliferation, stimulated the activation of inflammasome, and upregulated the production of mature interleukin (IL)-1β in macrophages via promoting miR-449a-5p/ATG4B-mediated autophagy [30].

The chondrogenic progenitor cells (CPCs) are a type of resident cells in cartilage with high chondrogenic differentiation potential and a strong ability to self-renew and possess a regenerative ability in both diseased and healthy articular cartilage tissues [70]. CPC-derived exosomes enhanced the proliferation and migration of chondrocytes and alleviated OA in a DMM mouse model, probably via upregulating miRNA 221-3p [48].

#### 2.2.3. Exosomes Derived from SFBs and Macrophages

Synoviocytes generally refer to SFBs and synovial macrophages, and SFBs are the major cell type in synovium [71]. Except for SMSC, SFBs and synovial macrophages also secret exosomes that regulate cartilage homeostasis and osteophyte formation [72]. Exosomes released by IL-1β treated SFB induced OA-like changes in articular chondrocytes by increasing the expression of catabolic genes, such as ADAMTS-5 and matrix metalloproteinases (MMP)-13, and downregulating the expression of anabolic genes, such as collagen type II (COL2) and aggrecan (ACAN) [71]. Exosomes from other synovial cells, including immune cells (e.g., macrophages, lymphocytes, and T cells) and endothelial cells, though not widely studied, are also believed to participate in the regulation of OA development [73]. Zeng et al. found that long non-coding RNA (lncRNA) prostate cancer gene expression marker 1 (PCGEM1) was overexpressed in exosomes from OA fibroblast-like synoviocytes (FLSs). FLS-derived exosomal PCGEM1 aggravated IL-1β-caused apoptosis and cartilage matrix degeneration in chondrocytes by sponging miR-142-5p and upregulating RUNX2 [49]. FLS-derived exosomal lncRNA H19 enhanced cell migration and proliferation, inhibited matrix degradation as well as alleviated OA progression by suppressing the miR-106b-5p/TIMP2 axis [74]. Though cytokines produced by macrophages and the imbalance between M1 and M2 macrophages are critical in OA pathogenesis, the effects of macrophage-derived exosomes on OA have been rarely studied thus far [75].

#### 2.2.4. Exosomes Derived from Osteoblasts and Osteocytes

The remodeling of subchondral bone is a critical feature of OA and strongly associated with disease severity and joint pain in clinical OA patients [76]. Altered crosstalk between articular cartilage and the subchondral bone, which can be modulated by exosomes in OA progression, has attracted much attention but not been well studied. Wu et al. found that exosomes produced by osteoblasts in osteoarthritic, sclerotic subchondral bone contained a high level of miR-210-5p, which decreased the rate of oxygen consumption by chondrocytes, altered their bioenergetic state, and accelerated the progression of cartilage degeneration [32]. Exosome-like EVs have been extracted from osteoblasts harvested from OA subchondral bones. The OA osteoblast-derived exosomes were found to have upregulated expression of five miRNAs—hsa-miR-885-3p, hsamiR-4717-5p, hsamiR-210-5p, hsa-miR-135a-3p, and hsa-miR-1225-5p—than those obtained from the healthy controls; the physiological and pathological roles of these molecules still remain unclear [19]. 

Osteocytes release miRNA-containing exosomes, which deliver their components via blood circulation to the recipient cells to regulate biological processes [77]. In addition, osteocytes are sensitive to mechanical strains. Cultured under cyclic stretch of 8% shape variable at a frequency of 0.1 Hz for 30 min, osteocytes produce exosomes containing differentially expressed miRNAs compared with those from non-loading groups. These exosomes promoted the proliferation and osteogenesis of human PDLSCs by activating the miR-181b-5p/PTEN/AKT signaling pathway [78]. Myostatin, a myokine secreted by muscles, suppressed the expression of miR-218 in osteocyte-derived exosomes. Treated with these exosomes, osteoblasts showed decreased osteoblastic differentiation and down-regulated activity of the Wnt signaling pathway [79]. Osteocyte exosomes were also found to accelerate benign prostatic hyperplasia development by promoting cell proliferation [80].

#### 2.2.5. Exosomes Derived from Adipose Tissue

IPFP is intraarticular adipose tissue that functions to reduce mechanical loading and absorb shock, and act as an abundant source of cytokines, lipid mediators as well as regenerative cells for cartilage repair [81]. IPFP is primarily comprised of adipocytes, and other cell types, including IPFP-derived MSCs and immune cells, are also found. As discussed earlier, intense interest has been spurred in IPFP-derived MSCs and IPFP-Exos [65].

Given the regulatory roles of adipose tissue in immune and nonimmune functions, compositional and functional analyses of adipocyte-derived exosomes can provide valuable information on the communications between adipocytes and other cells, such as immune cells, in the joint. A proteomic analysis of exosomes from obese diabetic and obese non-diabetic rats has been conducted. Among the 509 proteins identified, 200 of them were differentially expressed [82]. Sano et al. characterized the proteomic profiles of exosomes obtained from differentiated 3T3-L1 adipocytes and found that hypoxic culture upregulated the total protein amount in the exosomes and enriched the enzymes related to de novo lipogenesis [83]. According to Kita et al., adipose-derived exosomes can function as signaling packages and waste disposal bags [84]. Several lines of evidence support the role of adipose-derived exosomes in modulating macrophage polarization and hence inflammation [85,86,87]. Considering that obesity is a major risk factor for OA, investigations into adipose-derived exosomes may shed light onto molecular mechanisms underlying OA pathogenesis and the concurrent crosstalk between joint tissues.

#### 2.2.6. Exosomes Derived from PRP

Blood-derived products, including plasma- and serum-based whole blood derivatives, have been applied to OA treatment via IA injection for years [88]. IA injection of PRP has been reported to promote the proliferation and differentiation of chondrocytes and facilitate matrix synthesis [89]. Three types of platelet granules have been defined: dense granules, α-granules, and lysosomes, and they differ in size, content, biomarker, synthesis process, and function [90]. Extracting exosomes from other types of granules is mainly based on size and specific membrane proteins [91]. Previous studies showed that exosomes originating from platelets were sufficient to enhance anabolic marker expression and prevent the release of proinflammatory cytokines in chondrocytes derived from OA patients, showing the same regulatory effects as the full blood product [51]. In addition, the therapeutic effects of PRP-derived exosomes in inhibiting apoptosis and promoting proliferation of chondrocytes were achieved by activating the Wnt/β-catenin signaling pathway [17]. The PRP-derived exosomes are relatively easy to prepare, do not require cell culture, and have minimal risks of disease transmission, making PRP-derived exosomes highly promising in OA treatment. 

#### 2.2.7. Exosomes Derived from Other Cells

Exosomes derived from vascular endothelial cells (EC-Exos) were found to promote the progression of OA; EC-Exos increased the susceptibility of mouse chondrocytes to anoxidative stress by inhibiting p21 expression and autophagy, leading to more apoptotic chondrocytes in the mouse OA model [29]. The serum of OA patients was found to have elevated levels of T cell-derived, CD3- and CD4-positive exosomes, and platelet-derived EVs positive for annexin V and CD61^+^ and negative for CD45, as compared to that of healthy controls [92,93]. Exosomes from immune cells, such as B cells, T cells, and dendritic cells, caused the production of several cartilage-degrading enzymes (including MMP-1, MMP-3, MMP-9, and MMP-13) and inflammatory cytokines and chemokines (including IL-6, IL-8, monocyte chemoattractant protein (MCP)-1, and MCP-2) in SFBs from OA patients [94,95,96,97]. Exosomes from tenocytes were found to facilitate the tenogenic differentiation of MSCs, promoting the healing of injured tendons and increasing the maximum loading and ultimate stress in tendons [53]. Cyclic stretch force-induced PDL cells secreted exosomes that suppressed the production of IL-1β by inhibiting the NF-κB pathway in macrophages [54]. Human PDL fibroblast (hPDLFs)-derived exosomes induced inflammation and inhibited osteogenesis by osteoblasts [55]. Static compressive force stimulated the production of exosomes in PDLFs. These exosomes, containing a high level of the Yes-associated protein (YAP), promoted macrophage polarization toward the M1 phenotype [56]. Research on exosomes derived from these cells is just the beginning. More research is needed on their roles in OA pathogenesis and treatment.

## 3. Extraction, Bioengineering Modification, and Delivery of Exosomes

**Figure 3:**
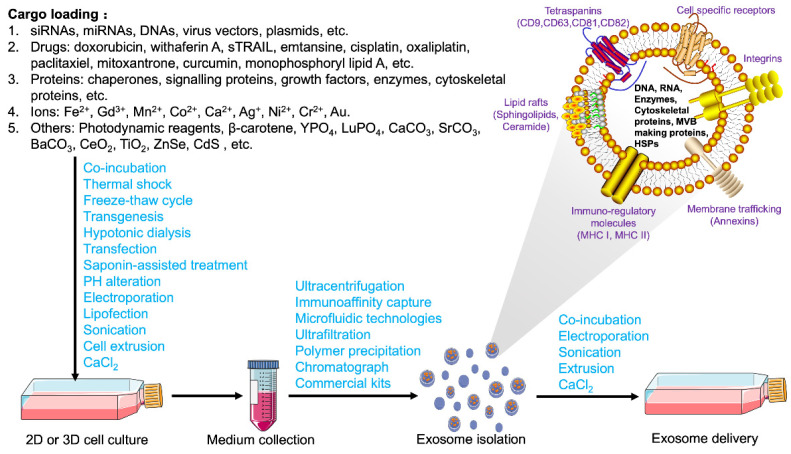
Cargo loading, isolation, and delivery strategies for engineered exosomes. Bioactive molecules, such as nucleic acids, vectors, plasmids, drugs, ions, and other compounds were added in the cell culture medium. Exogenous cargo can be loaded into exosomes by several methods, such as electroporation, lipofection, sonication, and CaCl_2_ treatment. Cells loaded with exogenous cargo secreted exosomes containing these bioactive molecules into cell culture medium. Cells expressing target peptides by plasmid transfection produce exosomes that can target specific cell populations. These engineered exosomes were isolated and purified from the culture medium via different methods. Through co-incubation or other strategies, exosomes loaded with endogenous and/or exogenous cargo can be taken up by recipient cells for the regulation of gene expression and cell function.

EVs are heterogeneous, cell-secreted membranous structures, which can be classified into exosomes, microvesicles, and apoptotic bodies based on biogenesis, size, and release pathways [98]. Depending on the intrinsic functions and conditions of source cells, unique protein profiles are exhibited by exosomes derived from different cells [99]. Due to the similarities between different kinds of EVs, it is vital to isolate and identify high-purity exosomes to understand their biological functions and elucidate their mechanisms of action. In addition, naturally occurring exosomes have several drawbacks, such as insufficient targeting ability and efficacy. Therefore, bioengineering processes are required to overcome these limitations. Figure 3 depicts the general steps of cargo loading, isolation, and delivery strategies for engineered exosomes, which are discussed thoroughly in the following sections.

### 3.1. Extraction, Identification, and Storage of Exosomes

Conditioned cell culture media are the most common source for exosome collection. Different methods based on the physical, chemical, and biological properties of exosomes have been developed to optimize the extraction, but standard operation procedures have not been established. Ultracentrifugation, immunoaffinity capture, ultrafiltration, size-exclusion chromatograph, charge neutralization-based polymer precipitation, and microfluidics-based techniques are commonly used methods for exosome extraction [100]; various precipitation- and column-based exosome isolation kits have also been developed (Figure 3) [101]. Whether a certain method or a combination of different methods should be selected depends on sample properties and research objectives. Whichever methods are applied, the goal for extraction remains the same, i.e., to maximize yield and purity while minimizing changes in protein content, size distribution, and surface charge during extraction. An in-depth discussion of different collection methods is beyond the scope of this article. Detailed extraction processes have been elaborated thoroughly in the published literature [102,103]. Several publications discussed the strengths and weaknesses of different methods to extract, characterize, and purify exosomes, and the selection of the most appropriate method(s) depends on the application and origin of exosomes [23,100,103,104].

There are two major types of exosome characterization methods: external characterization and inclusion characterization [105]. External characterization refers to the examination of morphology and particle size. Transmission electron microscopy (TEM) and scanning electron microscopy (SEM) are common methods for observing exosome morphology. SEM reveals the exosome surface microstructure, while TEM shows the internal structure and morphology of exosomes [106]. Nanoparticle tracking analysis (NTA) technology is applied for measuring the concentration and size of exosomes. Inclusion characterization is generally employed to detect membrane proteins, lipid rafts, and phospholipids present in the lipid bilayer, which can be detected by dynamic light scattering (DLS), flow cytometry, and western blotting [105]. Exosomes exhibit unique protein and lipid profiles that reflect the nature of donor cells and could be used as biomarkers for exosome identification. Common protein components include cytoskeletal proteins (e.g., actin), heat shock proteins (e.g., Hsp70 and Hsp90), bioactive proteins (e.g., GTPases, annexins, and flotillin), cytosolic proteins (e.g., GAPDH), antigen presentation proteins (major histocompatibility complex (MHC)-I, -II), tetraspanin membrane proteins (e.g., CD9, CD63, CD81, and CD82), proteins involved in multivesicular body biogenesis (e.g., Alix and TSG101), and vesicle trafficking (e.g., Tsg101) [19]. Membrane lipids vary in different exosomes and include cholesterol, ceramide, sphingolipids, phosphoglycerides, glycolipid GM3, and glycerophospholipids with saturated fatty-acyl chains [107]. Besides the methods described above, DLS, tunable resistive pulse sensing, and atomic force microscopy can also be employed to identify exosomes [108].

The preservation technologies of exosomes mainly include cryopreservation, freeze-drying, and spray-drying. Low temperature helps to maintain the quantity and contents of exosomes. It is recommended by the International Society of Extracellular Vesicles that exosomes be suspended in phosphate buffered saline and stored at −80 °C [109]. The addition of permeable and non-permeable antifreeze protects exosomes from ice crystal formation inside the vesicles and the imbalanced osmosis in the freezing process [110]. Freeze-drying, as a widely used method for preserving heat-sensitive materials, can dehydrate and dry exosomes at low temperatures under vacuum conditions [111]. Lyophilized exosomes can be stored at room temperature and conveniently reconstituted without affecting their pharmacokinetics [112]. Unlike lyophilization, which requires three continuous stages, spray drying is a single-step process. It is more economical but brings the risk of changing exosomal morphology [113]. Compared with cell-based therapies, the storage conditions of exosomes are generally less strict. Besides, frozen cells require recovery and function restoration prior to their clinical application, making them less convenient and more time consuming to handle compared to exosomes [114,115].

### 3.2. Contents and Loading Strategies for Exosomes

The constituent molecules of exosomes, including nucleic acids, lipids, proteins, and metabolites, differ in different exosomes, depending on the biogenesis mechanism, the cellular origin, developmental phase, environment, and epigenetic modification [116]. In vitro intracellular exosome loading during exosome biogenesis can be achieved by changing the culture condition and gene expression of the origin cells. For example, physical factors, such as low intensity pulsed ultrasound, moderate mechanical stress, and hypoxia have been found to convert exosomal contents to a chondroprotective mode [117,118,119]. Pretreatment with pharmacological agents, such as curcumin and kartogenin, enhances the exosomes’ ability to induce chondrogenesis by stem cells, promotes chondrocyte proliferation, and facilitates the repair of articular cartilage defects [64,120,121]. Biological factor-treated exosomes, such as those pretreated with transforming growth factor (TGF)-β1 or modified with activated transcription factor 4 mRNA, protect cartilage and alleviate OA progression by promoting the M2 polarization of synovial macrophages and inducing autophagy, respectively [122,123].

Genetic alteration is another widely used method to change exosomal content and function. miRNAs incorporated in exosomes participate in the intercellular communication in osteoarthritic joints [124]. The SF of OA patients was found to have upregulated miRNAs such as miR-155-3p, miR-16-2-3p, miR-504-3p, and miR-210-5p, and downregulated ones including miR-6878-3p, miR-146a-5p, and miR-26a-5p [125]. Available techniques to load therapeutic RNA mimics into exosomes include co-transfecting producer cells with plasmids, viruses, or bicistronic vectors, electroporating cells to facilitate the migration of small RNAs, and transient transfection with commercially available transfection reagents [126]. By using genetically modified parent cells, therapeutic agents were integrated into the corresponding exosomes [127]. Usually, non-coding RNA such as microRNA and lncRNA are induced to overexpress in the parent cells. These cells, usually MSCs, secrete exosomes containing high levels of desired RNAs that perform different roles in OA progression according to the gene properties [128]. Thus far, genetically modified cells have been employed to generate exosomes that are chondroprotective, anti-inflammatory, anti-apoptosis, and promote chondrocyte proliferation and migration [33,129,130]. 

The widely applied ex vivo extracellular exosome loading strategy refers to directly co-incubating exosomes with therapeutic agents and mixing them under appropriate conditions (Figure 3). For example, doxorubicin was successfully loaded onto pancreatic stellate cell-, pancreatic cancer cell-, and macrophage-derived exosomes via co-incubation [131]. Mixed with milk-derived exosomes, paclitaxel was loaded on the vesicles and showed significant therapeutic effects with low systemic and immunologic toxicities [132]. Co-incubation requires no special equipment, possesses high reproducibility, and does not compromise the integrity of exosome membrane structure. However, because of the relatively low loading efficiency, a large quantity of therapeutic agents is required [23]. To improve the drug loading efficiency on exosomes, electroporation, thermal shock, ultrasonic treatment, freeze-thaw cycles, transgenesis, pH gradient method, extrusion, hypotonic dialysis, transfection, and saponin-assisted treatments are applied to the synthesis process and show promising results (Figure 3) [133,134,135,136].

### 3.3. Bioengineered Modification and Delivery Strategies of Exosomes 

Exosomes can be taken up by cells via endocytosis, direct membrane fusion, and pinocytosis [137]. The endocytic pathway is the main route by which exosomes enter the cell, release the contents, and exert their biological effects. However, direct delivery of exosomes, such as IA or subcutaneous injections, is associated with quick clearance in vivo and limited effective period [138]. Chondrocyte-targeted drug delivery is even more challenging due to the biological barrier formed by a dense matrix of proteoglycans, collagen, and highly negatively charged glycosaminoglycans in the cartilage [66], which requires more exosomes in a higher concentration. To improve yield, elongate retention time, and optimize treatment effects, several strategies have been proposed and studied, such as the development of exosome-mimetic nanovesicles (EMNVs), alteration of the culture condition, membrane surface modification, and controlled release with biomaterial platforms [103,139].

As mentioned above, appropriate cell culture conditions promote the production of exosomes. For example, UC-MSCs grown in 3D microcarrier-based scaffolds yielded 20-fold more exosomes than 2D cultures. If combined with tangential flow filtration (TFF) for exosome extraction, the production of exosomes could be further improved 7-fold more than 3D cultures [140]. A rotary cell culture system (RCCS) simultaneously provides shear stress, hydrostatic pressure, and buoyancy force, creating an environment of microgravity that benefits cell adhesion, proliferation, and aggregation; exosome secretion by UC-MSCs was significantly promoted at 36 rpm/min within 196 h [42]. EMNVs are another method to achieve a large-scale production of exosomes. The generation of EMNVs via serially extruding cells through micro-sized filters boosted the yield of exosomes by over 100 folds and kept the biological functions similar to naïve exosomes [141,142]. When applying EMNVs, attention should be paid to the changed lipid species as well as altered membrane compositions compared with naïve exosomes, as such changes may affect the PK/PD behavior of EMNVs in vivo [143].

Several strategies modifying exosomal surface structures have been put forward to improve the entry of exosomes to cells that might be applied in OA studies. For example, chondrocyte-targeting exosomes were prepared by fusing the lysosome-associated membrane glycoprotein 2b (Lamp2b) protein present on the exosome surface with the chondrocyte-affinity peptide (CAP). These exosomes effectively encapsulated miR-140 and specifically entered chondrocytes to deliver the cargoes in vitro [47]. Equipping exosomes with cell-penetrating peptides (CPPs), such as arginine-rich CPPs (e.g., octa-arginine peptides, oligoarginine peptides, and human immunodeficiency virus type 1 Tat (48–60) peptide), facilitated exosome entry into the cell by stimulating cell micropinocytosis [144]. Coating exosomes with the amphiphilic cationic CHP (cCHP) nanogel particles is a polymer-based surface engineering method to facilitate exosome content delivery and increase the encapsulation of large-size nucleic acids (e.g., plasmid) [145]. One issue concerning hybrid exosomes is their similar cytotoxicity as liposomes (Lipofectamine). Therefore, further investigation is required to develop liposomes with less toxicity [146]. Increasing the efficiency of fusion between exosomes and the targeted cells is another approach. Studies have shown that an increased fusion efficiency between recipient cells and exosomes was achieved by enhancing membrane rigidity by enriching cholesterol and sphingolipid [138]. Vascular stomatitis virus (VSV)-G protein, when harbored on the surface of fusogenic exosomes, facilitates the delivery of membrane proteins into the target cell membranes in vitro and in a mouse intramuscular injection model [147]. The integration of exosomes with connexin 43 also promotes direct cytoplasmic transfer of exosome payloads [148].

Biomaterials are applied for exosome encapsulation and sustained-delivery, to extend the half-life of exosomes and augment their therapeutic effects [149]. Human joints that may be affected by OA are enclosed in the joint capsule (Figure 1). Therefore, IA injection of exosomes is preferable, as it is safer than the systematic application and has a low risk of side effects. By virtue of their affinity and compatibility with cartilage, several kinds of bioengineered hydrogel scaffolds have been applied to optimize the delivery of exosomes to cartilage, such as photoinduced imine-crosslinking hydrogel glue [150], chitosan hydrogel [151], light triggerable hyaluronic acid hydrogel [152], alginate-based hydrogel [153], ECM/gelatin methacrylate composite scaffolds [36], and a highly adhesive hydrogel, the AD/CS/RSF/EXO hydrogel (alginate-dopamine, chondroitin sulfate, regenerated silk fibroin, and exosome hydrogel) [154].

**Figure 4:**
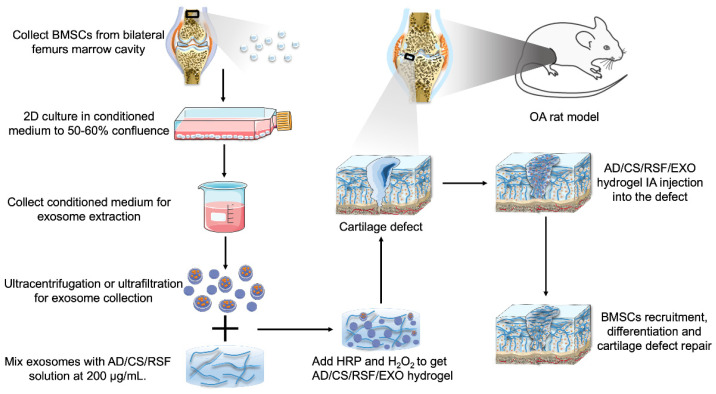
Schematic of fabricating AD/CS/RSF/EXO hydrogels for cartilage defect repair in a rat OA model. BMSCs were aseptically isolated from the bilateral femur marrow cavities of male Sprague-Dawley (SD) rats. When the cells reached 50–60% confluency in 2D culture flasks, they were rinsed and incubated for 48 h in serum-free medium. The collected conditioned medium was ultracentrifuged and ultrafiltered to obtain exosomes. The exosomes were mixed with AD/CS/RSF pre-gel solution, and then H_2_O_2_ and HRP were added to induce gelation. Subsequently, the cartilage defect was filled with the exosome-containing adhesive hydrogel. The exosomes released by the hydrogels recruited BMSCs that migrated and infiltrated the hydrogel and promoted BMSC proliferation and differentiation into chondrocytes. By inducing ECM production and neo-cartilage formation, the hydrogel facilitated the regeneration of cartilage defect in situ.

Processes for hydrogel-based scaffold preparation and delivery are similar among different kinds of hydrogels. Take the recently designed AD/CS/RSF/EXO hydrogel as an example [154]. As shown in Figure 4, exosomes extracted from the BMSCs-conditioned medium were mixed with the AD/CS/RSF pre-gel solution at 200 μg/mL. Then, horseradish peroxidase (HRP) and H_2_O_2_ were added to initiate crosslink formation and form a hydrogel. Subsequently, 500 μL AD/CS/RSF/EXO hydrogel containing 100 μg exosomes were injected into the cartilage defect of a rat knee joint via a syringe. The injected hydrogel quickly formed in situ and conformed to the defect shape within 3s. Covalent bonds formed between the amine and sulfhydryl groups on the surface of surrounding ECM and the chemical residues of the hydrogel (e.g., phenolic hydroxyl groups, *N*-hydroxysuccinimide, and catecholamine). As a result, the hydrogel generated adhesive binding with the surrounding native cartilage tissue due to the formation of covalently crosslinked networks. Besides, the loaded exosomes could be sustainedly released by the hydrogels, with around 87.51% of the encapsulated exosomes released into phosphate-buffered saline over 14 days. Exosomes released from hydrogels recruited BMSCs to scaffold implantation sites, promoted the proliferation and differentiation of MSCs, and accelerated ECM remodeling and cartilage defect regeneration. Hydrogel-based scaffolds are advantageous in controlled exosome release and operable for injection therapy under arthroscopy.

## 4. In Vivo Efficacy of Exosomes for OA Treatment

Considerable advances in exosome-based therapies have been demonstrated in several disease models [16]. However, exosomes have not been utilized in OA-related studies until recent years. Therapeutic effects, such as pain relief [34], cartilage defect repair [155], subchondral bone protection [35], and synovitis amelioration [30], have been observed in OA research. The delivery method of exosomes for in vivo OA treatment reported thus far has only been intra-articular injection.

Exosomes derived from MSCs and other sources have been tested in vivo to evaluate their therapeutic effects in OA treatment. Used in an MIA-induced rat OA model, exosomes obtained from BM-MSCs effectively enhanced cartilage repair, ECM synthesis, and joint pain relief [34]. IPFP-MSC-derived exosomes also prevented cartilage damage and alleviated gait abnormality in a mouse OA model by maintaining cartilage homeostasis [44]. PRP-Exos decreased the apoptotic rate of OA chondrocytes and decreased the OARSI (Osteoarthritis Research Society International) score of cartilage samples from OA joints of rabbit models [17]. SFBs overexpressing miR-126-3p generated exosomes that suppressed cartilage degeneration and inflammation in an OA rat model [50]. CPC-derived, exosome-containing EVs enhanced the repair of articular cartilage in a surgically induced mouse OA model and stimulated chondrocyte migration and proliferation in vitro via upregulating miRNA 221-3p [48]. Such beneficial effects have been attributed to the role of exosomes in regulating different signaling pathways, such as mTOR, Wnt/β-catenin, YAP, and non-coding RNAs (Table 1). Besides, treatment of MSCs with engineered exosomes showed enhanced joint-protective effects in OA animal models. For example, by fusing the exosomal membrane protein, Lamp 2, with MSC-binding peptide E7, engineered exosomes (E7-Exo) could be employed in the targeted delivery of kartogenin, a small heterocyclic molecule, to synovial fluid-derived MSCs (SF-MSCs). E7-Exos induced in vitro and in vivo differentiation of SF-MSC into chondrocytes. Furthermore, co-intra-articular injection of SF-MSCs together with E7-Exo in the knee joints showed superior therapeutic effects compared to SF-MSC injection alone in a rat OA model [121].

## 5. Discussion

Mediating intercellular communications, exosomes have demonstrated therapeutic potential in the diagnosis and treatment of various diseases and can be harnessed in OA-related studies. Published research has confirmed that for OA patients, the production and contents of exosomes from chondrocytes, synovial fluid, and serum are largely changed [156]. Besides, the exosomes derived from aging chondrocytes were found to transmit senescence-associated characteristics to adjacent cells and hinder their chondrogenic abilities [157].

At present, disease-modifying therapeutic options for OA are rather limited, warranting future explorations and investigations into potential disease-modifying treatment regimens. Emerging as a trending research area, exosomal therapy has attracted much attention due to its good biocompatibility as well as unique regulatory roles in immunity, inflammation, senescence, tumorigenesis, etc. The pathogenesis of OA is closely related to inflammation and aging. Therefore, injecting bioengineered exosomes or modifying native cell-produced exosomes to regulate the joint microenvironment and related cell function is potentially beneficial for OA prevention and treatment.

Exosomes derived from different types of cells regulate and influence the functions of recipient cells in different ways. Previous studies on the beneficial effects of exosomes in OA treatment focused on exosomes derived from only one cell source. The observed beneficial or adverse effects and potential regulatory mechanism of exosomes from different origins have been illustrated. OA is a degenerative disease of the whole joint, and multiple types of cells and tissues are involved in OA initiation and progression. The intra-articular environment is particularly complex and dynamic. Therefore, using exosomes derived from different cell types to simultaneously target different cells and tissues of the joint could be a promising approach worth investigating in future studies. For example, exosomes isolated from several cell sources exhibited chondroprotective effects. The combined application of exosomes produced by BM-MSC, ADSC, and synovial fibroblasts can potentially show synergistic effects on OA treatment as they target different major cell types in the joint.

Although results from preclinical studies have confirmed the chondroprotective effects of bioengineered exosomes, investigations into the efficacy of exosomes for OA treatment are still in their early stages. To optimize and extend the application of exosomes in OA diagnosis and treatment, several issues should be taken into consideration in future studies. First, the average pore size in the articular cartilage ECM is estimated to be around 6.0 nm [158]. Only small cationic nanocarriers, usually with a diameter smaller than 15 nm, can overcome this biological barrier [159]. Considering that the diameter of exosomes ranges between 30–150 nm, it is important to increase the delivery efficiency of exosomal contents to chondrocytes. Besides, the thickness of cartilage considerably affects the delivery of exosomes. In vivo tests of exosomes conducted to date mostly employed small animals such as mice, rats, and rabbits. The cartilage thickness of these animal models is significantly lower than human cartilage (~50 µm in mice, 100–150 µm in rats, and 350–700 µm in rabbits compared to 1500–2000 µm in humans) [160]. In addition, most in vitro studies were conducted in cultured chondrocytes instead of full-thickness cartilage explants, limiting the applicability of the results to in vivo scenarios. Existing extraction methods are limited by the low exosome yield, posing a major challenge to the clinical applications of exosomes. Undesired RNAs (e.g., retroviral genomes) or proteins unintentionally incorporated in exosomes, as well as off-target delivery, are also issues that need to be carefully considered. In addition, although encapsulating exosomes within a scaffold is a feasible option to achieve controlled release of exosomes and reduce the number of injections needed [161], material pharmacokinetics and possible toxicity should be carefully evaluated. Due to a lack of effective methods to separate exosomes from the other two kinds of EVs, it remains a challenge to explicitly elucidate the functions and physiochemical properties of exosomes. Besides, extracting homotypic exosomes with consistent contents is critical for precision therapy and minimum side effects caused by unintended by-products. In addition, rational designs of exosome delivery tools require a further understanding of the mechanisms responsible for exosomes targeting recipient cells and the binding affinities. Lastly, it is unclear in some cases how or why exosomes derived from different cells have varying biological activities. Therefore, a future research avenue is to figure out the active factors in various exosomes and their potential mechanisms of action in OA treatment.

The quick turnover of synovial fluid in the joint and the rapidly decreased transport efficacy into cartilage with increasing thickness necessitate strategies for enhancing exosome uptake to maximize the therapeutic effects of exosomes on chondrocytes, which reside deep within the dense, anionic cartilage matrix [162]. Previous studies reported approaches to overcoming the biological barrier of cartilage and improving the delivery efficacy of drugs and biomolecules. For example, controlling the surface charge of exosomes to achieve desirable electrostatic interactions with ECM could be a promising strategy to enhance drug penetration and transport through the full thickness of cartilage [163]. Functionalizing polyamidoamine (PAMAM) dendrimer nanocarriers with poly(ethylene glycol) (PEG) improved the tissue binding ability, penetration depth, and residence time of PAMAM dendrimer [159]. It was found that this modified dendrimer, when conjugated with insulin-like growth factor 1 (IGF-1), penetrated bovine cartilage with comparable thickness to humans’ within 2 days and significantly enhanced the retention of therapeutic IGF-1 within rat knees [159]. Another method to deliver large-sized therapeutics is via cationic peptides and proteins [164,165,166]. These studies indicate that it is feasible, albeit difficult, to overcome the biological barrier formed by cartilage ECM for effective exosome delivery.

It is worth noting that most in vivo tests of exosomes were conducted in small animals, including mouse, rat, and rabbit models. To date, no large animal studies or human clinical trials have been completed to evaluate exosomal treatment of OA. An ongoing clinical trial (ClinicalTrials.gov NCT04719793) evaluates the efficacy of umbilical cord-derived Wharton’s jelly (UC-WJ) for knee OA treatment. While exosomes are present in UC-WJ, it also contains various other components, such as hyaluronic acid, cytokines, growth factors, and other EVs [167,168]. The benefits of exosomes alone, therefore, will be unknown in this clinical trial. Few animal studies conducted thus far described the safety of exosomal treatment of OA, probably because unlike other pharmacological agents, exosomes are cell-secreted products and less likely to be toxic. Besides, exosomes are usually injected locally into the articular cavity, which is much safer than systematic administration. Therefore, a safety assessment of exosomal treatment is not as crucial as testing other OA drugs. Nevertheless, future studies are recommended to bridge this knowledge gap. Currently, insufficient evidence from preclinical research and clinical trials significantly hinders the translation of exosomal therapies from basic research to clinical applications. However, as the promising therapeutic effects of exosomes are being revealed in more basic research, an increasing number of large animal tests and clinical trials can be expected in the future. In conclusion, though faced with challenges, exosome-based therapies are promising in OA diagnosis and treatment and worthy of further investigations. 

## Figures and Tables

**Table 1:** Summary of major findings of OA-related studies involving the use of exosomes.

Cells	Source	Extraction	Dose	Delivery Method	Target Cells	Results	Ref
VECs	Conditioned medium	Ultrafiltration	100 μg	Co-incubation for 24 h	Primary chondrocytes	Promoted OA progression by inhibiting chondrocyte autophagy, downregulating p21 expression, and increasing ROS production and apoptosis.	[29]
OA chondrocytes	Culture supernatant	Ultracentrifugation	1 × 10^6^/mL	Co-incubation	Synovial macrophages	Promoted OA progression by stimulating inflammasome activation and upregulating mature IL-1β production in synovial macrophages	[30]
Primary chondrocytes	Conditioned medium	Ultracentrifugation	200 μg/mL	Co-incubation for 48 h Intra-articular injection	Chondrocytes	Prevented OA via the restoration of mitochondrial function and macrophage polarization toward the M2 phenotype	[31]
OA osteoblasts	Conditioned medium	Ultracentrifugation	20 μg/mL	Co-incubation for 14 d	Chondrocytes	Promoted OA progression by suppressing oxygen consumption by chondrocytes via miR-210-5p.	[32]
BM-MSCs	Conditioned medium	Ultracentrifugation	10 μg/mL	Co-incubation for 24 h	Chondrocytes	Promoted proliferation and inhibited apoptosis of chondrocyte via miR-206/GIT1 axis	[33,34]
BM-MSCs	Conditioned medium	Ultracentrifugation	250 ng	Intra-articular injection	Chondrocytes	Prevented OA development by inhibiting the degradation of cartilage and the formation of osteophyte	[35]
BM-MSCs	Conditioned medium	Ultracentrifugation	200 μg/mL	3D printed ECM/GelMA/exosome scaffolds	Osteochondral defect rabbit model	Prevented OA development by facilitating cartilage regeneration and restoring chondrocyte mitochondrial function	[36]
SMSCs	Conditioned medium	Ultracentrifugation	5 μg	Co-incubation for 12 h	Chondrocytes	Prevented the development of OA by facilitating migration, proliferation and ECM secretion and suppressing chondrocyte apoptosis	[37]
SMSCs	Conditioned medium	Ultracentrifugation	10^10^ particles	Intra-articular injection	DMM mice model	Prevented OA development by enhancing cartilage tissue regeneration via miR-140-5p upregulation of Wnt and YAP	[38]
ESC-MSCs	Conditioned medium	Ultrafiltration	5 μg/mL 100 μg	Co-incubation for 48 h Intra-articular injection	TMJ condylar chondrocytes	Prevented OA development via inflammation attenuation and matrix homeostasis restoration	[39]
ESC-MSCs	Conditioned medium	Ultracentrifugation	881 ng	Intra-articular injection	DMM OA model	Prevented OA development by balancing cartilage ECM synthesis and degradation	[40]
iPSC-MSCs	Conditioned medium	Ultracentrifugation	8 μL 10^10^/mL	Intra-articular injection	Collagenase-induced OA model	Prevented OA development by promoting migration and proliferation of chondrocytes	[41]
UC-MSCs	Conditioned medium	Ultracentrifugation	10 μg/mL 100 μg	Co-incubation for 72 h Intra-articular injection	Rat cartilage defect model	Mechanical stimulation increased the expression level of LncRNA H19 in exosomes, which promoted chondrocyte proliferation, matrix synthesis, and inhibited apoptosis	[42]
ADSCs	Conditioned medium	Ultracentrifugation	400 µg/mL	Co-incubation for 48 h	Chondrocytes	Prevented OA development by promoting chondrogenesis and suppressing inflammation via upregulating miR-221 and miR-145	[43]
ADSCs	Conditioned medium	Ultracentrifugation	10^8^ particles	Intra-articular injection	DMM and MIA induced OA model	Prevented OA development by inhibiting proteoglycan degradation and cartilage destruction and ameliorating gait abnormality	[44,45]
AFSC	Conditioned medium	Precipitation	30 μg 100 μg	Co-incubation for 72 h Intra-articular injection	MIA-induced OA mice model	Prevent the development of OA by promoting chondrocyte proliferation, cartilage matrix synthesis, and polarizing macrophages to M2 phenotype	[46]
Engineered CAP-Lamp2b exosomes	Conditioned medium	Ultracentrifugation	10 μg 100 μg	Co-incubation for 3 h Intra-articular injection	Chondrocytes DMM OA rat model	Prevented OA development by delivering miR-140 to deep cartilage regions and inhibiting cartilage-degrading proteases	[47]
CPCs	Conditioned medium	Ultracentrifugation	10^8^/mL 8 × 10^7^ particle	Co-incubation for 3 h Intra-articular injection	Chondrocytes	Enhanced articular cartilage repair by stimulating chondrocyte proliferation and migration via upregulating miRNA 221-3p	[48]
Synoviocytes	Conditioned medium	Ultracentrifugation	20 μg/mL	Co-incubation for 24 h	Chondrocytes	Promoted OA progression by inducing apoptosis and cartilage matrix degradation via upregulating miR-142-5p/RUNX2	[49]
Synovial fibroblasts	Patient synovial fluid	Ultracentrifugation	2 × 10^9^/mL 20 μg	Co-incubation for 48 h Intra-articular injection	ACLT + MMx OA rat model	Prevented OA development by suppressing chondrocyte apoptosis, constraining inflammation, and cartilage degeneration	[50]
PRP	PRP	exoEasy Maxi Kit	50 μg/mL 100 μg/mL	Co-incubation for 24 h Intra-articular injection	Chondrocytes	Prevented OA development by facilitating proliferation and reducing apoptosis of chondrocyte via Wnt/β-catenin	[17]
CPRP	Whole blood	Ultracentrifugation	1.42 × 10^9^ particles	Co-incubation for 48 h	OA chondrocytes	Prevented OA development by inducing chondrogenic gene expression changes and preventing proinflammatory cytokine release	[51]
IPFP	IPFP	Ultracentrifugation	10 μL 10^10^/mL	Intra-articular injection	DMM mice model	Prevented OA development by alleviating articular cartilage damage via miR-100-5p downregulation of mTOR	[44]
Tenocyte	Conditioned medium	Ultracentrifugation	486.3 μg/mL	Co-incubation for 48 h	Tendon stem cells	Promoted tendon healing by regulating tendon ECM metabolism and inducing the tenogenic differentiation of MSCs via upregulating transforming growth factor-beta	[52,53]
Periodontal ligament cells	PureExo^®^ exosome isolation kit	Precipitation	5 μg/mL	Co-incubation for 48 h	Macrophage	Regulated macrophage function and maintained inflammation homeostasis by suppressing IL-1β via inhibiting NF-κB signaling pathway	[54]
LPS-pretreated PDLFs	Conditioned medium	Ultracentrifugation	100 μg/mL	Co-incubation for 48 h	Osteoblast	Prevented bone remodeling by inducing inflammation and inhibiting osteogenic activity of osteoblasts, promoting macrophage polarization toward M1 via YAP	[55,56]

VECs: vascular endothelial cell; BM-MSCs: bone marrow mesenchymal stem cells; ESC-MSCs: embryonic stem cell-derived MSCs; iPSC-MSCs: induced pluripotent stem cells-derived MSCs; UC-MSCs: umbilical cord mesenchymal stem cells; CPCs: chondrogenic progenitor cells; DMM: destabilization of the medial meniscus; ACLT + MMx: anterior cruciate ligament and resecting the medial menisci; PRP: platelet-rich plasma; CPRP: citrate-anticoagulated platelet-rich plasma; SMSCs: synovial mesenchymal stem cells; IPFP: infrapatellar fat pad; AFSC: amniotic fluid stem cells; ADSCs: adipose-derived stem cells; MIA: monosodium iodoacetate; PDLSCs: periodontal ligament-derived stem cells; PDLFs: periodontal ligament fibroblasts.

## Data Availability

Not applicable.
